# Identification and characterization of the lncRNA signature associated with overall survival in patients with neuroblastoma

**DOI:** 10.1038/s41598-019-41553-y

**Published:** 2019-03-26

**Authors:** Srinivasulu Yerukala Sathipati, Divya Sahu, Hsuan-Cheng Huang, Yenching Lin, Shinn-Ying Ho

**Affiliations:** 1 Institute of Bioinformatics and Systems Biology, National Chiao Tung University, Hsinchu, Taiwan; 2 Institute of Biomedical Informatics, Center for Systems and Synthetic Biology, National Yang-Ming University, Taipei, Taiwan; 3 Bioinformatics Program, Taiwan International Graduate Program, Institute of Information Science, Academia Sinica, Taipei, Taiwan; 4 Interdisciplinary Neuroscience Ph.D. Program, National Chiao Tung University, Hsinchu, Taiwan; 5 Department of Biological Science and Technology, National Chiao Tung University, Hsinchu, Taiwan; 6 Center For Intelligent Drug Systems and Smart Bio-devices (IDSB), National Chiao Tung University, Hsinchu, Taiwan

## Abstract

Neuroblastoma (NB) is a commonly occurring cancer among infants and young children. Recently, long non-coding RNAs (lncRNAs) have been using as prognostic biomarkers for therapeutics and interventions in various cancers. Considering the poor survival of NB, the lncRNA-based therapeutic strategies must be improved. This work proposes an overall survival time estimator called SVR-NB to identify the lncRNA signature that is associated with the overall survival of patients with NB. SVR-NB is an optimized support vector regression (SVR)-based method that uses an inheritable bi-objective combinatorial genetic algorithm for feature selection. The dataset of 231 NB patients that contains overall survival information and expression profiles of 783 lncRNAs was used to design and evaluate SVR-NB from the database of gene expression omnibus accession GSE62564. SVR-NB identified a signature of 35 lncRNAs and achieved a mean squared correlation coefficient of 0.85 and a mean absolute error of 0.56 year between the actual and estimated overall survival time using 10-fold cross-validation. Further, we ranked and characterized the 35 lncRNAs according to their contribution towards the estimation accuracy. Functional annotations and co-expression gene analysis of LOC440896, LINC00632, and IGF2-AS revealed the association of co-expressed genes in Kyoto Encyclopedia of Genes and Genomes pathways.

## Introduction

Neuroblastoma (NB) is the most common cancer in children, comprising 10% of all childhood cancers^1^. Most cases occur in very young children under the age of one year^2^; hence, NB is commonly referred to as an embryonic tumour^3^ and is responsible for approximately 11% of cancer deaths in children. Initially, the tumour originates in tissues of the sympathetic nervous system and is thus found as lesions in the adrenal glands, pelvis or abdomen chest^4^. The characteristics of neoplasms are highly enigmatic because these tumours exhibit either spontaneous regression or rapid progression. The prospect of survival depends on the age at diagnosis, tumour stage, and genetic features. According to The International Neuroblastoma Staging System, NB is staged into five groups: stage 1 to 4 and 4S based on metastasis formation and lymph node involvement^5,6^. The treatment of NB exhibits clinical diversity; hence, the treatment response is correlated with clinical and biological factors, including cancer risk group, age, and genetic abnormalities. Children with stage 1 and stage 2 neuroblastomas can be cured with surgery alone as a primary therapy^7^. Infants with stage 4 neuroblastomas exhibit better prognosis in response to treatment with chemotherapy and surgery^8^. In contrast, patients with high-risk NB exhibit poor event-free survival after chemotherapy, whereas improved event-free survival is observed in patients with advanced-stage NB after radiotherapy and chemotherapy followed by autologous bone marrow transplantation^9^. Despite treatment conditions, only 40–50% of patients with NB exhibit long-term survival^10^. Due to the heterogeneous nature of NBs, the clinical behaviour and molecular mechanisms underlying tumour growth are largely unknown, and more efficacious therapeutics are necessary to control this cancer.

The most common genetic abnormality observed in NB is amplification of the MYCN gene in NB cells. MYCN-mediated oncogenic transformation is responsible for aggressive tumour formation and poor prognosis in NB^11^. Further, genetic abnormalities associated with NB include loss of heterozygosity at the distal short arm of chromosome 1, which is associated with clinical outcome^12,13^, hyperdiploid features^14^, and defects in the function of nerve growth factor (NGFR)^15,16^. Genome-wide studies have sought to identify protein biomarkers for improved NB therapies. For instance, pharmacodynamic biomarkers have been developed to evaluate the mechanism of PI3K/AKT/mTOR pathway signalling activity and MYCN protein expression in children with NB^17^. Expression of biomarkers, including X-linked inhibitor of apoptosis and vascular growth factors, regulates bone marrow metastasis in NB^18^. Genomic amplification of the MYCN oncogene is associated with NB tumour aggressiveness and poor prognosis in NB patients^19^. Germline mutations in the anaplastic lymphoma kinase gene are largely responsible for familial NB, and this germline mutation can serve as potential therapeutic target for NB^20^. Although advances in treatment conditions and therapeutics have improved patient prognosis, long-term survival of the high-risk group has not been considerably improved. Hence, the identification of potential targets associated with NB survival is urgently required.

Over the past several years, advancements in next-generation sequencing (NGS) and microarray technologies have increased the interest in non-coding RNAs (ncRNAs), including small non-coding RNAs, such as miRNAs, piRNAs, and snoRNAs, and long non-coding RNAs (lncRNAs), given their significant roles in specific diseases. In particular, the role of lncRNAs in evolution and genome function is a newly described phenomenon. LncRNAs are non-coding RNAs that are >200 nucleotides in length and have been implicated in pathological and biological process through post-transcriptional regulation of mRNA processing and cis regulation^21^. Over the last decade, several studies have identified that lncRNAs play a significant role in several biological processes^22^. LncRNAs are highly stable and easily detectable in body fluids^23,24^. Several studies have revealed the significance of lncRNAs in various cancers. For instance, specific lncRNAs are up- or down-regulated in prostate cancer; lncRNAs, such as PCGEM-1, PCAT-1, and PCA3, play critical roles in prostate cancer^25,26^. The lncRNA HOTAIR is up-regulated, silences genes through interactions with LSD1 and PRC2 and is also involved in protein degradation via interaction with E3 ubiquitin ligases in various cancer types, include lung, ovarian, and pancreatic cancers^27–29^. LncRNAs also play important roles in NB. Specifically, ncRNA possess oncogenic properties, and its overexpression is correlated with poor prognosis in NB patients^30^. Overexpression of NDM29 in NB cell lines is associated with chemosensitivity^31^. Despite of advances in RNA-sequencing technologies, functions of several lncRNAs are not yet validated. LncRNAs are emerging as crucial players in tumorigenesis by directly or indirectly acting as tumor suppressors^32^ or oncogenes^33^. Various approaches were developed to use lncRNAs as potential targets in cancer, such as post-transcriptional targeting of lncRNAs^34^, modulation of lncRNAs using genome-editing techniques^35^, and loss of lncRNA function by inhibition of RNA-protein interactions using RNA-binding small molecules^36^. The identification of lncRNA signature in the context of cancer provides an opportunity to explore lncRNAs as possible targets and improve our knowledge of lncRNAs association with the overall survival of NB.

Several researchers have attempted to predict NB patient survival. Oberthuer *et al*. predicted individual survival rates for NB patients using the automatic relevance determination (CASPAR) algorithm^37^. Wei *et al*. developed a survival predictor using an artificial neural network and identified 19 genes that predict clinical outcome in NB patients^38^. Gene-wide promoter methylation profiling and cox elastic net analysis were utilized to predict NB patient outcome, and the degree of methylation of retinoblastoma 1 (RB1) and teratocarcinoma-derived growth factor 1 (TDGF1) was associated with poor survival^39^. MicroRNA expression profiling and support vector machines (SVMs) were used to predict event-free survival in NB patients^40^. However, few studies exist that use lncRNAs for survival prediction in NB patients. Divya *et al*. utilized lncRNA expression profiles and reported that SNHG1 is highly expressed and significantly associated with poor survival in NB patients^41^. Another study by Divya *et al*. used lncRNA expression data from 493 NB patients and identified a 16-lncRNA prognostic signature that predicts event-free survival^42^. In addition, lncRNA expression profiling was also used in other cancer types for prediction purposes. The lncRNA signature was used to predict the overall survival in esophageal squamous cell carcinoma^43^. Six lncRNAs were identified which significantly correlate with the disease free survival in patients with colorectal cancer^44^. Zhu *et al*. identified a 24-lncRNA signature to predict the prognosis in gastric cancer^45^. Five lncRNAs were identified which significantly correlate with the prognosis of clear cell renal cell carcinoma^46^. Tu *et al*. utilized lncRNA expression profiling and a random survival forest algorithm to predict risk groups in lung cancer patients^47^. Zhou *et al*. identified four lncRNAs that were significantly associated with overall survival in multiple myeloma patients using multivariate Cox regression and stratified analysis^48^. Meng *et al*. identified four lncRNA genes using a random survival forest algorithm to predict survival in breast cancer patients^49^. Recently, Wang *et al*. identified nine immune-related lncRNA signature in patients with anaplastic gliomas^50^. Genome-wide analysis study on 419 patients with glioblastoma identified six lncRNAs, AC005013.5, UBE2R2-AS1, ENTPD1-AS1, RP11-89C21.2, AC073115.6, and XLOC_004803 which distinguished the high and low risk groups^51^. In conclusion, utilization of lncRNA expression in cancer survival prediction could aid in the understanding of the molecular mechanisms underlying cancer progression and the identification of potential biomarkers.

Accordingly, this study proposed the SVR-NB method to identify the lncRNA signature that is strongly associated with overall survival in NB patients. Different from our previous studies^41,42^, SVR-NB was developed based on support vector regression (SVR)^52^ and an inheritable bi-objective combinatorial genetic algorithm (IBCGA)^53^ to select a small set of lncRNAs as a signature among a large number of lncRNAs. We retrieved RNA-seq data and overall survival information of NB patients from the database of gene expression omnibus (GEO) accession GSE62564. In clinical research, the time to death is an event of interest; hence, we exclusively focused on patients who died from NB. After the filtration process, 104 patients with 104 expression profiles consisting of 783 lncRNAs and corresponding overall survival information were obtained for further analysis. SVR-NB identified 35 out of 783 lncRNAs which are strongly correlated with overall survival in NB patients. SVR-NB using 10-fold cross-validation (10-CV) achieved a mean squared correlation coefficient of 0.85 ± 0.009 and a mean absolute error of 0.56 ± 0.09 years between actual and estimated overall survival times in NB patients. We analysed the roles of identified lncRNAs in different cancers. Furthermore, functional annotation and co-regulated gene expression analyses of top ranked lncRNAs were discussed. We hope that these findings will improve multimodal therapy and survival in patients with NB.

## Results and Discussion

### Overall survival estimation

We utilized SVR-NB to identify the lncRNA signature that correlated with the overall survival in NB patients. We utilized 104 lncRNA expression profiles of 783 lncRNAs and the corresponding overall survival data from 104 NB patients. SVR-NB used the feature selection algorithm IBCGA to identify a small set of lncRNAs as a signature that influence overall survival of NB patients.

SVR-NB achieved a best squared correlation coefficient of 0.89 and a mean absolute error of 0.49 years between the actual and estimated overall survival time using 10-CV from 30 independent runs (Table 1). SVR-NB obtained a mean squared correlation coefficient of 0.85 ± 0.009 and a mean absolute error of 0.56 ± 0.09 years in NB patients. We measure the feature frequency score (FFS) for each of 30 independent runs of SVR-NB to select one robust feature set with the highest FFS. The obtained signature of 35 lncRNAs has the highest FFS of 7.86 indicating that each lncRNA appears 7.86 times on average in the 30 runs. The FFS values of 30 runs are given in Supplementary Fig. S1.

**Table 1:** Performance of SVR-NB.

Method	Features Selected	Squared correlation coefficient	Mean absolute error (years)
SVR-NB	33	0.89	0.49
SVR-NB(Mean)	30.26	0.85 ± 0.009	0.56 ± 0.09
SVR-NB(FFS)	35	0.84	0.63
Ridge regression	783	0.62	0.87
LASSO	41	0.68	0.78
Elastic net	44	0.67	0.81

**Figure 1:**
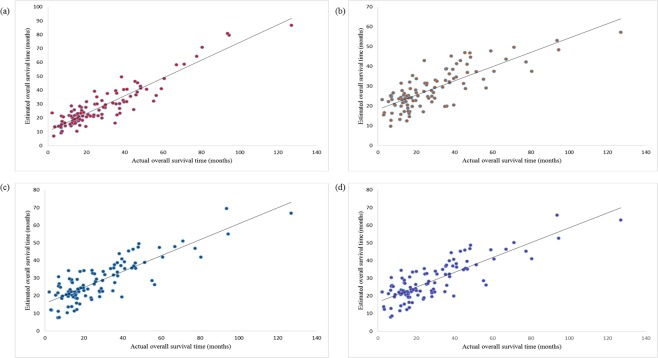
(**a**) Estimation performance of SVR-NB. (**b**) Estimation performance of ridge regression. (**c**) Estimation performance of LASSO regression. (**d**) Estimation performance of elastic net regression. X-axis refers to actual overall survival time, and Y-axis refers to estimated survival time.

We compared the SVR-NB method with three standard linear regression methods: ridge, LASSO and elastic net regression methods. Ridge regression used all the features and obtained a squared correlation coefficient of 0.62 and a mean absolute error of 0.87 years between the actual and estimated overall survival times. LASSO identified 41 features and achieved a squared correlation coefficient and a mean absolute error of 0.68 and 0.78 years, respectively. The elastic net method identified 44 features and obtained a squared correlation coefficient and a mean absolute error of 0.67 and 0.81 years, respectively, between the actual and estimated survival time. The SVR-NB estimation performance is better than that of these three standard regression methods. The correlation plots of SVR-NB, ridge, LASSO, and elastic net are presented in Fig. 1.

Additionally, we used the signature of 35 lncRNAs and Naïve Bayes classifier^54^ to classify the 352 NB patients into high risk and low risk groups. Naïve Bayes classifier achieved a leave-one-out cross-validation accuracy, Matthews correlation coefficient, precision, recall and area under ROC curve of 86.64%, 0.73, 0.86, 0.86, and 0.94 respectively. The prediction performance of Naïve Bayes classifier was evaluated using a receiver operating curve (ROC), as shown in Supplementary Fig. S2.

### SVR-NB validation

We evaluated the performance of SVR-NB in an independent test cohort of 127 patients with NB who are still living. The independent test cohort exhibits the mean overall survival time of 39.22 ± 15.42 months, whereas the predicted mean overall survival time is increased compared with the actual mean overall survival time 43.55 ± 17.58 months. The predicted mean overall survival time of 73 (50.96 ± 18.45) among the 127 patients is increased compared with the actual mean overall survival time (32.77 ± 16.18). The obtained squared correlation coefficient was 0.31 between actual overall survival time and predicted overall survival time.

**Figure 2:**
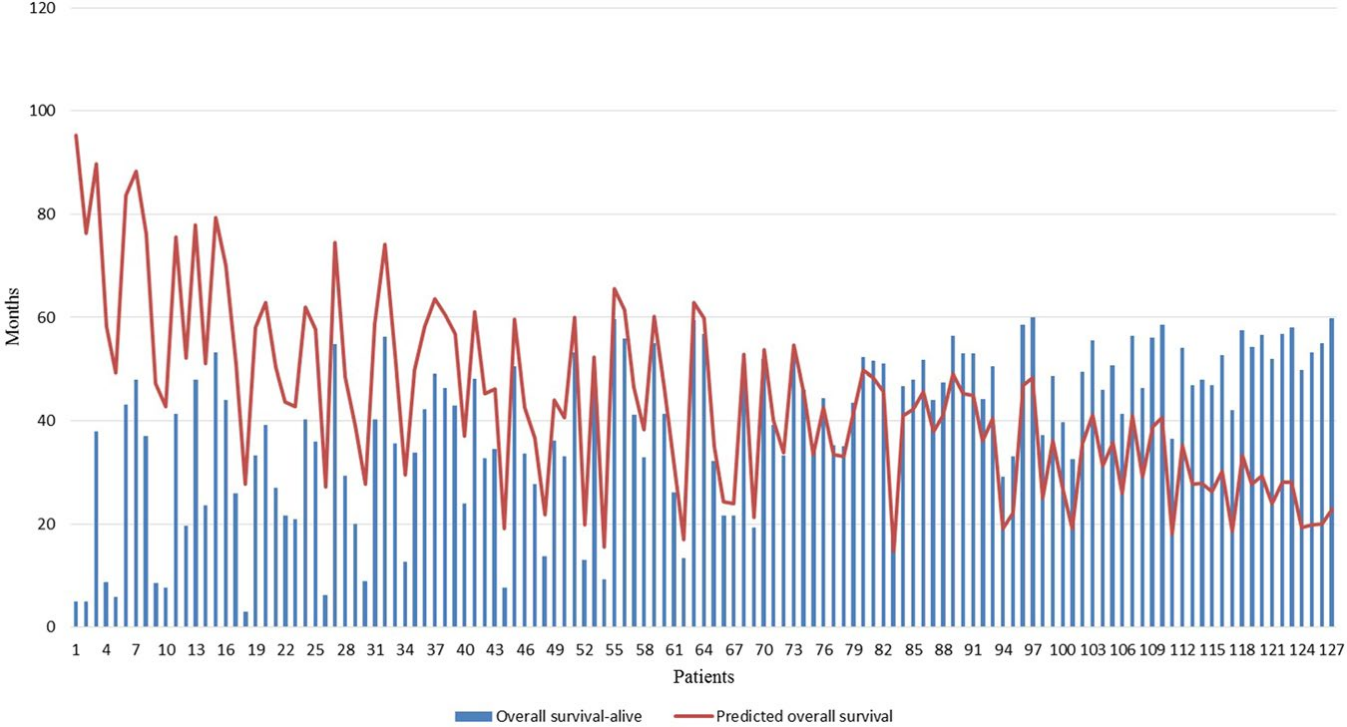
SVR-NB validation using an independent test cohort of 127 NB patients.

The prediction error in terms of mean absolute error for the remaining 54 patients whose predicted overall survival time is smaller than the actual overall survival time, which is 1.19 years between the actual overall survival time and predicted overall survival time. Comparing to the prediction error of 0.63 years obtained for the 104 NB patients using SVR-NB (FFS), whereas the prediction error of 1.19 years is higher, due to the small sample size. However, SVR-NB would perform better by increasing the training sample size. The estimation of overall survival in the independent test cohort is presented in Fig. 2.

### Ranking of the lncRNA signature

We ranked the lncRNAs of the identified signature using main effect deference (MED) analysis^55^. MED analysis reveals the contribution of each lncRNA among the lncRNA signature towards estimation accuracy of the overall survival time. LncRNAs with higher MED scores indicate a greater contribution of these lncRNAs towards the estimation accuracy of overall survival time, while the lncRNAs with lower MED scores indicates the lesser contribution. The top 10 ranked lncRNAs based on the MED analysis are LOC440896, LOC729770, LINC00632, CXCR2P1, LOC643542, LOC387720, IGF2-AS, DUX4L3, HAS2-AS1, and LINC01606. We ranked all 35 lncRNAs, and their corresponding MED values are presented in Table 2. The top 10 lncRNAs and their chromosome locations are provided in Supplementary Table S1.

**Table 2:** MED ranking of lncRNAs.

Rank	Ref-Seq ID	LncRNA-Symbol	MED score
1	NR_015361	LOC440896	2.588
2	XR_108432	LOC729770	1.694
3	NR_028344	LINC00632	1.655
4	NR_002712	CXCR2P1	1.451
5	NR_033921	LOC643542	1.388
6	XR_109027	LOC387720	1.296
7	NR_028043	IGF2-AS	1.282
8	NM_001164467	DUX4L3	1.279
9	NR_002835	HAS2-AS1	0.983
10	NR_038235	LINC01606	0.981
11	NR_030171	MIR492	0.975
12	NR_027088	LOC284661	0.953
13	NR_002145	OR2L1P	0.945
14	NR_003503	GGT8P	0.925
15	XR_109271	LOC400511	0.857
16	NR_027284	LINC00602	0.811
17	NR_033942	ARHGEF34P	0.768
18	XM_001717149	LOC100130503	0.719
19	NR_027321	LINC00964	0.649
20	NR_002766	MEG3	0.614
21	NR_026816	PSORS1C3	0.589
22	NR_003187	NCF1C	0.511
23	XR_109119	LOC100129223	0.369
24	XR_111273	LOC100509445	0.300
25	NR_033400	CSNK1G2-AS1	0.290
26	NR_029965	MIR431	0.258
27	NR_024192	HILS1	0.255
28	NR_026766	MYCNOS	0.236
29	NR_038977	LINC01239	0.155
30	NR_073404	LOC441081	0.125
31	NR_037890	DNAJB8-AS1	0.107
32	NR_024119	LINC00244	0.106
33	NR_046173	LOC254896	0.102
34	XR_110545	LOC730376	0.088
35	XR_109597	GDF5OS	0.066

### Significance of top ranked lncRNA in cancers

#### LOC440896

Uncharacterized LOC440896 alias AL353608.3 is differently expressed in various cancers. Genome-wide analysis studies on 79 small cell lung cancer patients reported that AL353608.3 is up-regulated and differently expressed in lung cell carcinoma compared with that of normal cells with a log2-fold change of 3.2^56^. RNA-sequencing of cells derived from patients with juvenile idiopathic arthritis demonstrated that AL353608.3 was up-regulated in inflammatory cells with a log2-fold change of 5 compared with that of normal cells^57^. This lncRNA is actively involved in breast cancer cells, and expression of AL353608.3 is up-regulated in breast cancer cells compared with that of normal counterparts^58^. Additionally, AL353608.3 was down-regulated in blood platelets from patients with pancreatic adenocarcinoma with a log2-fold change of −4.2 compared with that in healthy samples^59^, and expression of this lncRNA expression is also involved in glioblastoma^59^.

#### LINC00632

Long intergenic non-protein coding RNA 632 (LINC00632) is implicated in several major cancers. For instance, LINC00632 expression was up-regulated in breast cancer cells with a log2-fold change of 5.2 compared with that in normal cells^58,60^. Up-regulation of LINC00632 was observed in prostate carcinoma cells with a log2-fold change of 4.8 compared with that in healthy cells^61^. Additionally, down-regulation of LINC00632 is significantly associated with different cancer types, such as non-small cell lung carcinoma^59^ and medulloblastoma^62^, and down-regulation of LINC00632 is frequently observed in glioblastoma^63,64^. In addition to cancer tissues, LINC00632 is highly expressed in normal brain tissue with a mean RPKM of 3.06 ± 1.54^65^.

#### LOC643542

Uncharacterized LOC643542 is highly expressed in human normal tissues and 27 other tissue types, such as fat, kidney and brain, with mean RPKM values of 0.29 ± 0.17, 0.15 ± 0.11, and 0.07 ± 0.14, respectively^65^. Genome-wide association studies revealed the association of LOC643542 with major depressive disorder^66^. A meta-analysis of 1110 major depressive disorder cases reported that LOC643542 is localized in the brain region and exhibits a higher number of single-nucleotide polymorphisms^66^. Genome-wide association studies further confirm the association of LOC643542 in bipolar disorder^67^ and hyperactivity disorder^68^.

#### IGF2-AS

RNA-sequence analysis study on breast carcinoma patients revealed that IGF2-AS is up-regulated in HER2 breast carcinoma cells with a log2-fold change of −4.1 compared with that in normal cells^58^. Down-regulation of IGF2-AS was also observed in amyotrophic lateral sclerosis^69^ and Down syndrome (trisomy 21)^70^ with log2-fold changes of −1.5 and −2.9, respectively, compared with those in normal cells. IGF2-AS expression was up-regulated in glioblastoma^71^ with a log2-fold change of 3 and in childhood brain tumour ependymoma^62^ with a log2-fold change of 1.3.

#### HAS2-AS1

HAS2-AS1 is frequently down-regulated in different cancer types. RNA sequencing of six tumour types revealed that HAS2-AS1 is down-regulated in various cancers^59^. HAS2-AS1 expression was down-regulated in breast carcinoma cells, pancreatic carcinoma, colorectal carcinoma, non-small cell lung carcinoma, and glioblastoma cells with log2-fold changes of −3.2, −2.6, −1.8, −1.2, and −1.2, respectively, compared with those in normal cells^59^. In addition, HAS2-AS1 up-regulation was also observed in glioblastoma cells with a log2-fold change of 3.5^71^.

#### LINC01606

LINC01606 is implicated in various cancers. RNA-sequence analysis on LINC01606 revealed that LINC01606 is up-regulated in triple-negative breast cancer cells and HER2-positive breast carcinoma cells with log2-fold changes of 5.4 and 2.8, respectively^58^. Up-regulation of LINC01606 was also observed in oesophageal adenocarcinoma^72^. LINC01606 was down-regulated in pancreatic adenocarcinoma^59^ and glioma^71^ with log2-fold changes of −5 and −3.5, respectively. RNA-sequencing studies on different tumour types revealed down-regulation of LINC01606 in hepatobiliary carcinoma, non-small cell lung carcinoma, and colorectal carcinoma with log2-fold changes of −2.7, −2.4, and −2.1, respectively^59^.

Few studies reported the remaining four lncRNAs (LOC729770, CXCR2P1, LOC387720, and DUX4L3) among the top 10 ranked lncRNAs, involved in NB and other cancers. Though, these four lncRNAs LOC729770, CXCR2P1, LOC387720, and DUX4L3 have few experimental validations in NB, their contribution towards the overall survival estimation is higher ranked second, fourth, sixth, and eighth respectively. Hence, these four lncRNAs are potential biomarkers of NB survival time to be further validated. We summarize the top 10 ranked lncRNAs and their role in cancer/disorder in Supplementary Table S2.

Though there were limited number of experimental validations on lncRNAs in NB, we reported some studies to support the association between the identified lncRNAs and cancer. A study using a real-time reverse transcriptase polymerase chain reaction assay (qPCR) and western blot analysis on NB cells revealed that MYCN expression was found to be up-regulated and associated with the NB stage^73^. Northern blot analysis on Wilm’s tumor samples reported that IGF2-AS was found to be up-regulated in Wilms’ tumor samples compared to the healthy samples^74^. A qPCR and Sothern blot analysis on hepatocellular carcinoma cells revealed that IGF2-AS can significantly restrain the malignant cells and may act as gene therapeutic target^75^. Up-regulation of HAS2-AS1 was observed in oral squamous cell carcinoma using qPCR and western blot analysis^76^. LNC00964 expression was found to be down-regulated in colorectal cancer using qPCR analysis^77^. The qPCR and western blot analyses revealed the up-regulation of MEG3 in pancreatic ductal carcinoma^78^, multiple myeloma^79^, and ovarian cancer^80^.

### Expression difference in amplified MYCN and non-amplified MYCN groups

The GEO database (GSE62564) included 401 patients with MYCN non-amplified disease and 92 patients with MYCN amplified disease. We measured expression levels of the top 10 ranked lncRNAs in MYCN amplified and MYCN non-amplified groups. We observed a slight difference in the expression of top ranked lncRNAs in MYCN amplified and MYCN non-amplified groups. Of the top 10 ranked lncRNAs, the mean expression of LOC440896, LOC729770, LINC00632, CXCR2P1, LOC643542, LOC387720, IGF2-AS, DUX4L3, HAS2-AS1, and LOC100507651 are 0.18 ± 0.35, 0.24 ± 0.32, 4.42 ± 3.59, 4.24 ± 4.91, 0.36 ± 0.36, 0.08 ± 0.09, 2.10 ± 5.4, 0.39 ± 1.95, 0.33 ± 0.44 and 0.12 ± 0.89, respectively, in the MYCN-amplified group and 0.11 ± 0.19, 0.32 ± 0.10, 3.59 ± 7.79, 4.91 ± 3.92, 0.36 ± 0.12, 0.09 ± 0.05, 5.47 ± 1.79, 1.95 ± 1.60, 0.44 ± 0.21 and 0.89 ± 0.10, respectively, in the MYCN-non-amplified group. Box-plot representations of lncRNA expression in the MYCN-amplified and MYCN-non-amplified group are presented in Supplementary Fig. S3.

Additionally, we performed the survival analysis of the top 10 ranked lncRNAs using Kaplan-Meir (KM) survival curves. We used median expression of the lncRNA as a threshold to classify lncRNA expression into high expression group and low expression group. The KM-survival curves were plotted for the top 10 ranked lncRNAs. The overall survival KM plots for the two groups were shown in Supplementary Fig. S4.

Six lncRNAs among the top 10 are differently expressed in various normal human tissues, such as lung, liver, ovary, brain, and other tissues. The expression levels of these six lncRNAs in different tissues are shown in Supplementary Fig. S5 using the human body map.

### Functional annotations of LOC440896, IGF2-AS, and DUX4L3

We examined the functional annotations of the top 10 ranked lncRNAs using Database for Annotations Visualization and Integrated Discovery tool (DAVID)^81^. Each lncRNA is associated with specific functional annotations. For instance, among the top 10 ranked lncRNAs, LOC440896 is associated with the sequence feature of the putative uncharacterized protein FLJ45355. IGF2-AS is associated with the putative insulin-like growth factor2 antisense gene protein and sequence variant. DUX4L3 is associated with compositionally biased regions Ala-rich and Arg-rich and DNA binding region. The lncRNA DUX4L3 is associated with various gene-ontology terms, including nitrogen compound metabolic process (GO:0006807), biosynthetic process (GO:0009058), regulation of biological process (GO:0050789), regulation of metabolic process (GO:0019222), cellular metabolic process (GO:0044237), and biological regulation (GO:0065007).

Furthermore, the UCSC_TFBS algorithm available from DAVID was used to identify protein interactions, including transcription factors with sets of target genes. Four out of the top10 ranked lncRNAs including CXCR2P1, HAS2-AS1, DUX4L3, and LOC440896, are involved in protein interactions and have functions related to transcription factors. We summarize the functional annotations associated with the top 10 ranked lncRNAs in Table 3.

**Table 3:** LncRNA and their predicted protein interactions.

ID	Gene Name	Species	UCSC_TFBS
3580	C-X-C motif chemokine receptor 2 pseudogene 1 (CXCR2P1)	Homo sapiens	AP1, AP4, AREB6, ARP1, CDP, CDPCR3, CEBP, CETS1P54, CP2, E47, GATA1, GATA3, GR, GRE, HEN1, HNF1, HTF, IK3, LUN1, MYOD, MZF1, NF1, NFAT, P300, PAX4, PAX5, SEF1, SRF, TAL1ALPHAE47, TAL1BETAE47, TAL1BETAITF2, TAXCREB, TCF11, YY1
594842	HAS2 antisense RNA 1 (HAS2-AS1)	Homo sapiens	AHR, AHRARNT, AML1, AP1, AP4, AREB6, ARNT, ATF, ATF6, BACH1, BACH2, BRACH, CART1, CDC5, CDPCR3HD, CEBP, CREB, CREBP1, CREBP1CJUN, E2F, E47, E4BP4, EGR3, EVI1, FOXJ2, FOXO3, FOXO4, FREAC3, FREAC4, FREAC7, GATA1, GFI1, GRE, HAND1E47, HEN1, HFH1, HFH3, HSF1, HSF2, HTF, IK2, IK3, LHX3, LMO2COM, LUN1, MEIS1BHOXA9, MYCMAX, MYOD, NFE2, NFKB, NFY, NKX25, NKX61, NMYC, OCT1, P300, PAX2, PAX4, PAX6, PBX1, PPARG, RFX1, S8, SOX5, SRY, STAT3, STAT5A, USF, XBP1, YY1, ZIC3
653548	double homeobox 4 like 3 (DUX4L3)	Homo sapiens	AP2REP, AREB6, CDPCR3HD, FOXO3, FREAC4, HSF2, OCT1, P53, PAX3, PAX5, SPZ1, TCF11MAFG
440896	uncharacterized LOC440896 (LOC440896)	Homo sapiens	CEBPB, EVI1, FOXJ2, FREAC2, GATA1, IK3, ISRE, NKX25, PAX3, RP58, TCF11MAFG, TST1

### Co-regulated gene network analysis of LOC440896, LINC00632 and IGF2-AS

**Figure 3:**
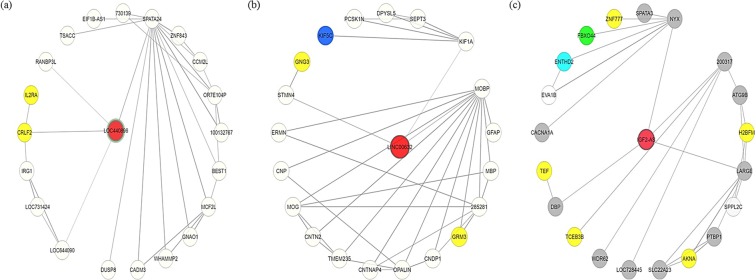
Co-expressed gene regulatory network of (**a**) LOC4408965, (**b**) LINC00632, and (**c**) IGF2-AS. Nodes represent lncRNAs, and edges represents coexpressed genes. The red node in the middle indicates the lncRNA. The yellow, blue, green, aqua and grey coloured nodes indicates the genes that are involved in different KEGG pathways.

We constructed the co-regulated gene network using COXPRESdb^82^ to identify gene coexpression relationships among the top ranked lncRNAs. We analysed coexpressed genes and their functions for the lncRNAs LOC440896, LINC00632 and IGF2-AS. Four coexpressed genes, including cytokine receptor-like factor 2 (CRLF2), spermatogenesis associated 24 (SPATA24), uncharacterized LOC644090 (LOC644090), and RAN binding protein 3-like (RANBP3L), are directly connected to LOC440896. CRLF2 and interleukin 2 receptor alpha (IL2RA) genes are involved in the Jak-STAT signalling pathway (KEGG ID: hsa04630) and cytokine-cytokine receptor interaction (KEGG ID: hsa04060). In the co-expressed gene network, LINC00632 is directly connected to stathmin-like 4 (STMN4), myelin-associated oligodendrocyte basic (MOBP) and kinesin family member 1A (KIF1A) genes. Three genes were identified in the co-expression network of LINC00632: G protein subunit gamma 3 (GNG3) and glutamate metabotropic receptor 3 (GRM3), which are involved in the glutamatergic synapse (KEGG ID: hsa04724), and kinesin family member 5C (KIF5C), which is involved in the dopaminergic synapse (KEGG ID: hsa04728). IGF2-AS is directly connected to like-glycosyltransferase (LARGE), nyctalopin (NYX) and the D site of albumin promoter (albumin D-box) binding protein (DBP) in the co-expressed gene network. The top 100 genes coexpressed with IGF2-AS are involved five different KEGG pathways, including platelet activation (KEGG ID: hsa04611), phospholipase D signalling pathway (KEGG ID: hsa04072), Rap1 signalling pathway (KEGG ID: hsa04015), cAMP signalling pathway (hsa04024), and endocrine resistance (KEGG ID: hsa01522). The three lncRNAs and the involvement of coexpressed genes based on KEGG pathway analysis is presented in Table 4. Gene co-expression networks for LOC440896, LINC00632 and IGF2-AS are presented in Fig. 3.

**Table 4:** KEGG pathway association of co-expressed genes for LOC440896, LINC00632, and IGF2-AS.

LncRNA	Gene symbol	Gene name	Correlation with lncRNA	KEGG Pathway name (KEGG ID)
LOC440896	SPATA24	spermatogenesis associated 24	0.42	▪ Jak-STAT signaling pathway (hsa05630). ▪ Cytokine-cytokine receptor interaction (hsa04060). ▪ Chagas disease (American trypanosomiasis) (hsa05142). ▪ Tuberculosis (hsa05152). ▪ Adrenergic signaling in cardiomyocytes (hsa04261)
	LOC644090	uncharacterized LOC644090	0.33	
	CRLF2	cytokine receptor-like factor 2	0.29	
	RANBP3L	RAN binding protein 3-like	0.19	
LINC00632	STMN4	stathmin-like 4	0.26	▪ GABAergic synapse (hsa04727) ▪ Morphine addiction (hsa05032) ▪ Retrograde endocannabinoid signaling (hsa04723) ▪ Neuroactive ligand-receptor interaction (hsa04080) ▪ Nicotine addiction (hsa05033)
	MOBP	myelin-associated oligodendrocyte basic	0.26	
	KIF1A	kinesin family member 1A	0.24	
IGF2-AS	NYX	nyctalopin	0.55	▪ Platelet activation (hsa04611) ▪ Phospholipase D signaling pathway (hsa04072) ▪ Rap 1 signaling pathway (hsa04015) ▪ cAMP signaling pathway (hsa04024) ▪ Endocrine resistance (hsa01522)
	LARGE	like-glycosyltransferase	0.52	
	DBP	D site of albumin promoter (albumin D-box) binding protein	0.46

Furthermore, we investigated the expression levels of these three lncRNAs in NB patients using integrated bioinformatics and wet-lab data analysis of NB data^83^, in which 88 human NB samples were analysed. Gene expression charts were generated using the gene expression activity chart plugin, which is available from the BioGPS gene annotation portal^84^. Expression charts for LOC440896, LINC00632 and IGF2-AS among 88 human NB samples are presented in Supplementary Fig. S6.

## Conclusions

Recent advances in NGS data have attracted considerable attention in the exploration of the significance of ncRNAs in cancer. LncRNAs are becoming a subject of interest in cancer research due to their critical role in multiple biological processes. Recent developments in computational biology and experimental techniques have identified thousands of lncRNAs in eukaryotes. However, only few lncRNAs are characterized and experimentally validated to confirm their disease association. Hence, developing computational models to identify the lncRNAs in cancer is an important task that would aid to understand the disease at lncRNA levels, and disease diagnosis. Various computational prediction models have been developed to discover non-coding RNAs and disease association^85–90^. Chen *et al*. developed potential computational models to identify the lncRNA and disease association^91,92^. Identification of the lncRNA signature associated with overall survival in cancer patients using well-validated computational methods is helpful for the therapeutic strategies. LncRNAs are implicated in tumorigenesis and exhibit diverse regulatory processes in cellular process. Thus, the identification of lncRNA signature would be important in terms of disease characterization and therapy. Therefore, we attempted to identify the lncRNA signature that is associated with the overall survival of NB patients, which could aid in NB therapeutics. Accordingly, we developed a survival time estimator called SVR-NB to estimate the overall survival time and identify the lncRNA signature that is associated with overall survival in NB patients. We incorporated the feature selection algorithm IBCGA into SVR to establish the optimized SVR model. SVR-NB identified a 35-lncRNA signature that is potentially correlated with the overall survival time of NB patients. SVR-NB obtained a 10-CV squared correlation coefficient of 0.85 ± 0.009 and a mean absolute error of 0.56 ± 0.09 years between the actual and estimated overall survival times in NB patients. In addition, SVR-NB performed better than standard regression methods, including ridge, LASSO and elastic net. Although, the estimation performance of SVR-NB is promising, it has some limitations due to the small sample size. The prediction error of SVR-NB on the independent test cohort was increased when compared to that on the training dataset. Nonetheless, SVR-NB performance can be improved by increasing the number of samples.

We ranked the lncRNAs of the identified signature based on their contribution towards the survival estimation. Furthermore, we analysed the roles of the top ranked lncRNAs in cancer. Functional annotations and co-regulated gene expression of LOC440896, LINC00632 and IGF2-AS are discussed. The expression levels of these three lncRNAs in NB samples were presented using expression charts. Although some of the lncRNAs among the top 10 ranked list, such as LOC729770, CXCR2P1, LOC387720, and DUX4L3 are uncharacterized, and not involved in NB, our analysis suggests that these four lncRNAs might exhibit critical roles in NB patients’ overall survival and are promising biomarkers of NB survival time for further validation.

The development of technologies for potential identification of lncRNAs and their role in cancer are important for NB diagnostics and therapeutics. Identified lncRNAs in this study could aid in the development of lncRNA-based targeted cancer therapies in NB patients.

## Materials and Methods

### Dataset

We retrieved the lncRNA expression dataset of 493 NB samples from GEO accession GSE62564. The details about preprocessing and normalization of the GSE62564 dataset is described in the work^41^. We applied filtration to the dataset, including elimination of duplicate entries, selection of samples who died from NB, and retrieval of overall survival time by using the sample ID. We eliminated samples with the overall survival time of less than 30 days. In the lncRNA filtration process, we applied log intensity variation^93^ to reduce the size of candidate features from 6260 to 783 lncRNAs. After the filtration process, the training dataset consisted of 104 patients with overall survival time and 104 expression profiles of 783 lncRNAs. Another dataset of 127 patients with NB who are alive from GEO accession GSE62564 was used as an independent test cohort.

### SVR-NB

This study proposed an overall survival time estimator SVR-NB based on SVR using IBCGA to identify the set of lncRNAs in NB patients. The functionality of SVR-NB is two-fold: to estimate the overall survival time and to identify significant lncRNAs strongly associated with overall survival.

The support vector machine (SVM) algorithm^94^, is useful in solving bioinformatics problems^95,96^. SVR is another version of SVM for regression. SVR has been widely applied in many biomedical fields, such as pharmaceutical research^97^ and cancer prognosis^98^. We have successfully applied an SVR incorporated with feature selection algorithm IBCGA for estimation of survival in patients with glioblastoma multiforme and lung adenocarcinoma^99,100^.

SVR-NB is developed based on ν-SVR for the given data points (x_*1*_, y_*1*_), … (x_*n*_, y_*n*_), where x_*i*_ ∈ R^*l*^ is an NB patient input sample and, y_*i*_ ∈ R^*k*^ is a target label (y_*i*_ is the overall survival time). The primal problem of ν-SVR is described as follows.

1 $${\rm{\min }}[\{\frac{1}{2}{w}^{T}(\varnothing ({x}_{i})+b)+C(\nu {\rm{\varepsilon }}+\frac{1}{m}\sum _{i=1}^{m}\,({\xi }_{i}+{\xi }_{i}^{\ast }))\}]$$

where ${\xi }_{i}\ge 0,\,\,{\xi }_{i}^{\ast }\ge 0,\,{\rm{\varepsilon }}\ge 0;\,{\rm{i}}=1,2,\ldots ,m$; and b is a constant.

Here, 0 ≤ *ν* ≤ 1, and *C* is the regularization parameter. The *ε*-insensitive loss function.

To avoid the over training, we used 10-fold cross-validation (10-CV) to evaluate the performance of the model. Pearson’s correlation coefficient (CC) was used as a fitness function. Pearson’s correlation coefficient (CC) is formulated as follows:

2 $$CC=\frac{{\sum }_{i=1}^{N}\,({x}_{i}-\bar{x})({y}_{i}-\bar{y})}{\sqrt{\lfloor {\sum }_{i=1}^{N}\,{({x}_{i}-\bar{x})}^{2}\rfloor [{\sum }_{i=1}^{N}\,{({y}_{i}-\bar{y})}^{2}]}}$$

where *x*_*i*_ and *y*_*i*_ are actual and estimated overall survival time of the *i*^th^ lncRNA respectively, and $\bar{x}$ and $\bar{y}$ are their corresponding means. Here, *N* is the total number of patients with NB. We used squared correlation coefficient to evaluate the model performance.

### Inheritable bi-objective combinatorial genetic algorithm

To select a minimal set of informative features from a large number of candidate features the inheritable bi-objective combinatorial genetic algorithm (IBCGA) is used. The IBCGA uses an intelligent evolutionary algorithm^101^ that can efficiently solve large parameter optimization problems. In this study, we propose a method for the identification of informative lncRNAs associated with NB overall survival based on the IBCGA and ν-SVR by maximizing the estimation performance in terms of correlation coefficient (CC). In this work, the LibSVM package^102^ was used for implementation of ν-SVR.

The encoded chromosomes and the customized IBCGA were designed as described in previous studies^99,100,103^. The chromosome of the IBCGA comprises 783 genes and three 4-bit genes for encoding γ, C, and ν for the ν-SVR. In this work, the parameter values are *r*_*start*_ = 10, *r*_*end*_ = 50, *N*_*pop*_ = 50, *P*_*c*_ = 0.8, *P*_*m*_ = 0.05, and *G*_*max*_ = 60^53^.

We evaluated the prediction performance using mean absolute error (*MAE*):

3 $$MAE=\frac{1}{N}\sum _{i=1}^{N}{|{y}_{i}-{x}_{i}|}^{2},$$

where *x*_*i*_ and *y*_*i*_ are actual and estimated overall survival time of the *i*^th^ lncRNA, respectively. Here, *N* is the total number of NB patients. The steps of IBCGA are as follows.

Step 1: (Initialization) Randomly generate a population of *N*_*pop*_ individuals.

Step 2: (Evaluation) Evaluate the fitness value of all individuals using the fitness function that is the squared correlation coefficient (S*CC*) in terms of 10-fold cross-validation (10-CV).

Step 3: (Selection) Use a tournament selection method that selects the winner from two randomly selected individuals to generate a mating pool.

Step 4: (Crossover) Select two parents from the mating pool to perform orthogonal array crossover operation.

Step 5: (Mutation) Apply a conventional mutation operator to the randomly selected individuals in the new population. Mutation is not applied to the best individuals to prevent the best fitness value from deterioration.

Step 6: (Termination test) If the stopping condition for obtaining the solution is satisfied, output the best individual as the solution. Otherwise, go to Step 3.

Step 7: (Inheritance) If r < *r*_*end*_, randomly change one bit in the binary genes for each individual from 0 to 1; increase the number r by one, and go to Step 3. Otherwise, stop the algorithm.

Step 8: (Output) Obtain a set of lncRNAs from the chromosome of the best individual.

### Ridge, LASSO and Elastic net

We compared three standard regression methods with SVR-NB. The Ridge regression is also called L2-penalized regression^104^. The Ridge regression conserves all the features to build prediction models. In the Ridge regression, the penalty term (λ) regularizes the coefficients of the predictors towards zero, if the coefficients take large values, and the optimization function is penalized. Hence, the Ridge regression shrinks the coefficients and reduces the model complexity. The least absolute shrinkage and selection operator (LASSO)^105^ was also employed to estimate the overall survival of NB patients. LASSO uses L1 regularization, in which some of the coefficients are neglected or regularized to zero for the evaluation of output^105^. Therefore, LASSO can help in the feature selection procedure. We chose λ (minimum λ) for the tuning parameter after 100 iterations of 10-CV. We used squared correlation coefficient and mean absolute error for the performance measurement.

Elastic net^106^ is an extension of the LASSO, in which LASSO and ridge regression are combined. The Elastic net method can be defined as follows

4 $$Min{}_{{\beta }_{0},\beta }(\frac{1}{2N}\sum _{i=1}^{N}\,{({y}_{i}-{\beta }_{0}-{x}_{i}^{T}\beta )}^{2}+\lambda {P}_{\alpha }(\beta )),$$

where *y*_i_ is the overall survival time at observation; *x*_*i*_ ∈ R^*m*^ is the vector of *m* lncRNA expression values for the *i*-th observation, *β*_0_ and *β* are regression coefficients, λ is a regularization parameter, and *N* is the total number of observations.

### Feature frequency score (FFS)

We measure the feature frequency score for each independent run as follows:

5 $$FFS=\sum _{i=1}^{{l}_{t}}\,f({z}_{i})/{n}_{t}$$

where *f*(*z*) is the feature frequency for feature *z* that presents in the lncRNA set, *n*_*t*_ is number of the features in the *t*-*th* signature, t = 1 …. R, and *Z*_*i*_ is the *i*-*th* lncRNA in the *t*-*th* solution.

## Supplementary information

Identification and characterization of the lncRNA signature associated with overall survival in patients with neuroblastoma

## Data Availability

All the data used in this analysis can be found at the database of gene expression omnibus (GEO) accession GSE62564.
