# Supplementary material for: Identification and characterization of the lncRNA signature associated with overall survival in patients with neuroblastoma
Source: Sci Rep. 2019 Mar 26;9:5125. doi: 10.1038/s41598-019-41553-y (PMC6435792; doi:10.1038/s41598-019-41553-y)
Supplement: Supplementary file 1 — Identification and characterization of the lncRNA signature associated with overall survival in patients with neuroblastoma [file 41598_2019_41553_MOESM1_ESM.docx]

**Identification and characterization of the lncRNA signature associated with overall survival in patients with neuroblastoma**

**Srinivasulu Yerukala Sathipati^1^, Divya Sahu^2^, Hsuan-Cheng Huang^2,3^, Yenching Lin^4^, and Shinn-Ying Ho^1,4,5,6*^**

^1^Institute of Bioinformatics and Systems Biology, National Chiao Tung University, Hsinchu, Taiwan

^2^Institute of Biomedical Informatics, Center for Systems and Synthetic Biology, National Yang-Ming University, Taipei, Taiwan

^3^Bioinformatics Program, Taiwan International Graduate Program, Institute of Information Science, Academia Sinica, Taipei, Taiwan

^4^Interdisciplinary Neuroscience Ph.D. Program, National Chiao Tung University, Hsinchu, Taiwan

^5^Department of Biological Science and Technology, National Chiao Tung University, Hsinchu, Taiwan

^6^Center For Intelligent Drug Systems and Smart Bio-devices (IDS^2^B), National Chiao Tung University, Hsinchu, Taiwan

*Corresponding author

Email address:

SYH: [syho@mail.nctu.edu.tw](mailto:syho@mail.nctu.edu.tw)

# **Supplementary Figures**

**Supplementary Figure S1.** Feature frequency score (FFS) plot. The highest FFS is 7.86 for the 23^rd^ run.


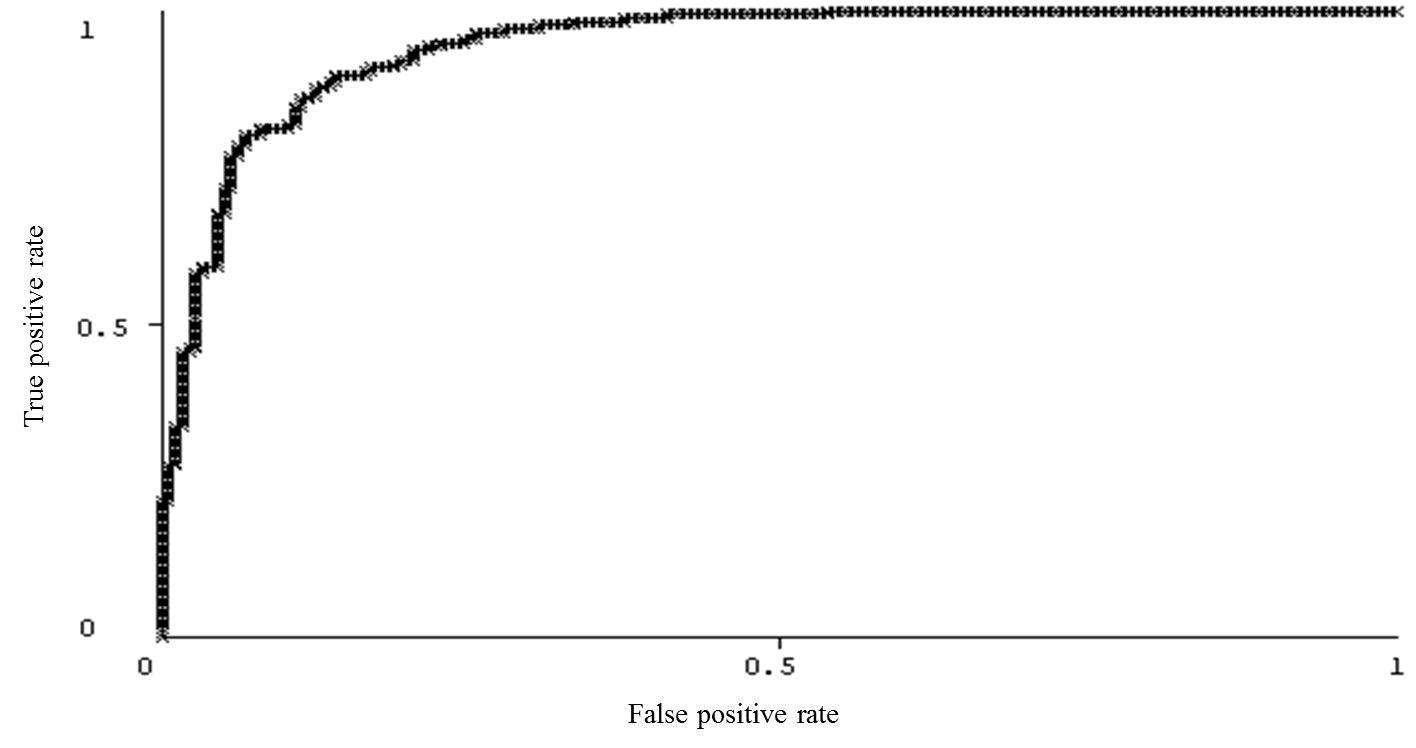


**Supplementary Figure S2**. Prediction performance of the Naïve Bayes classifier using a ROC curve. The area under ROC curve is 0.94.

**
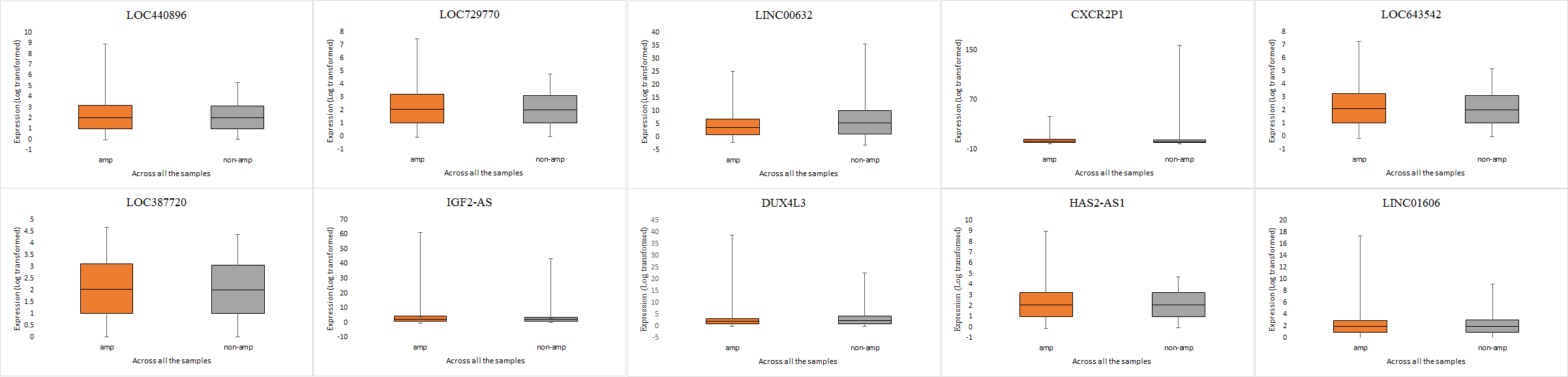
**

**Supplementary Figure S3.** Box-plot representation of the top 10 ranked lncRNAs in MYCN amplified and non-amplified samples.


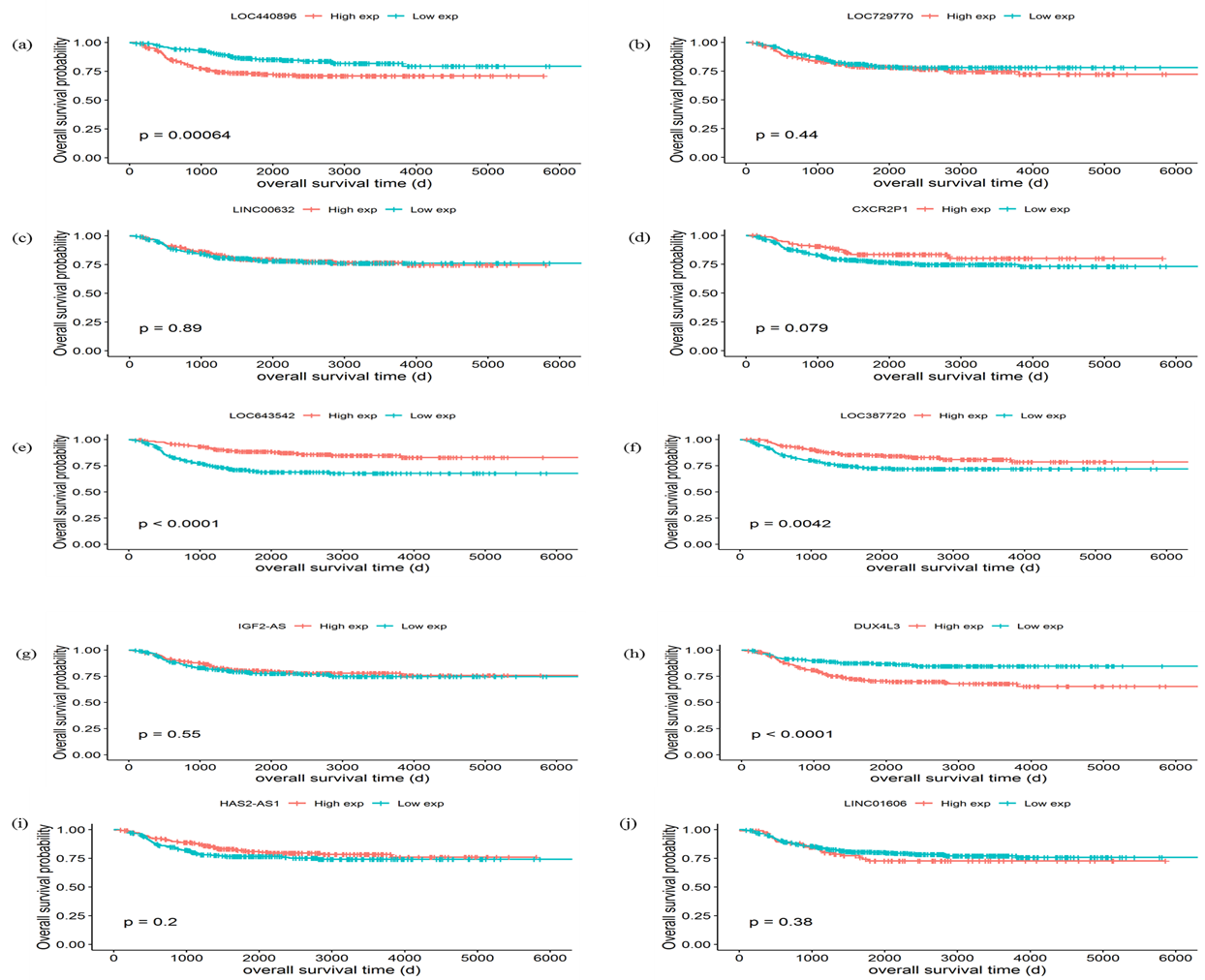


**Supplementary Figure S4**. Kaplan-Meier plots of (a) LOC440896, (b) LOC729770, (c) LINC00632, (d) CXCR2P1, (e) LOC643542, (f) LOC387720, (g) IGF2-AS, (h) DUX4L3, (i) HAS2-AS1 and (j) LINC01606 for the high expression and low expression groups.

**Supplementary Figure S5.** Six of the top 10 ranked expressed lncRNA levels in different normal tissues.


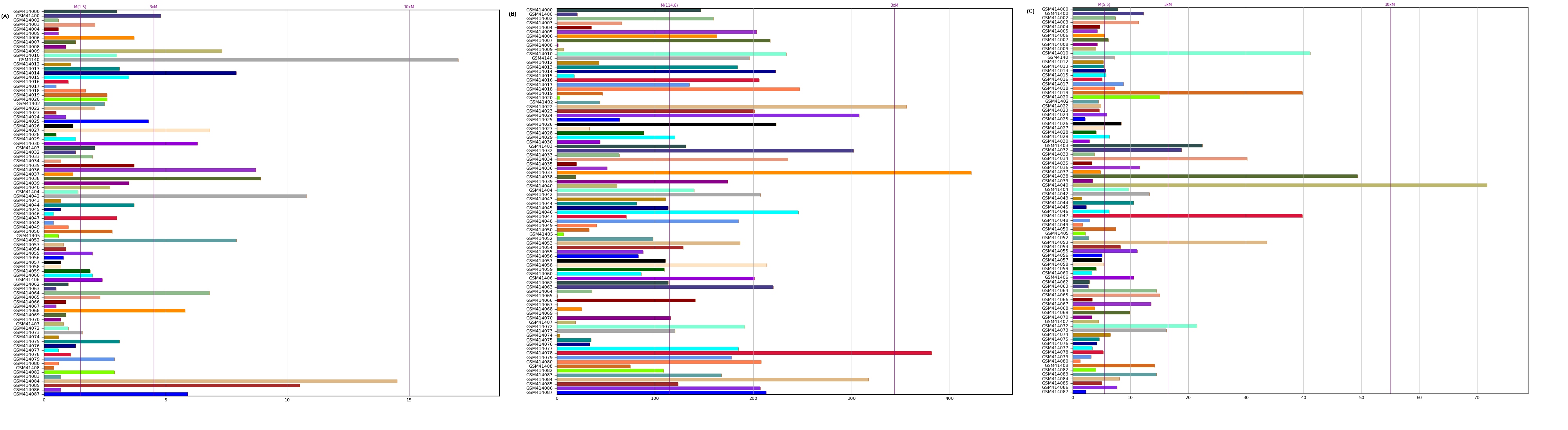


**Supplementary Figure S6**. **(A)** LOC440896 expression in 88 human neuroblastoma samples. **(B)** LINC00632 expression in 88 human neuroblastoma samples. **(C)** IGF2-AS expression in 88 human neuroblastoma samples obtained from GEO-GSE16476. LncRNA expression values in MAS5.0 signal intensity are reported on the X-axis, and neuroblastoma samples are indicated on the Y-axis. The median expression levels of LOC440896, LINC00632 and IGF2-AS are 1.5, 144.6 and 5.5 (MAS5.0 signal intensity values), respectively.

# **Supplementary Table**

**Supplementary Table S1.** Top 10 ranked lncRNAs and their chromosomal location.

| **Ref-ID** | **LncRNA** | **Gene Name** | **Chromosome** | **Cytoband** |
| --- | --- | --- | --- | --- |
| NR_015361 | LOC440896 | uncharacterized LOC440896 | 9 | 9p11.2 |
| XR_108432 | LOC729770 | uncharacterized LOC729770 | 2 | 2q36.1 |
| NR_028344 | LINC00632 | long intergenic non-protein coding RNA | X | Xq27.1 |
| NR_002712 | CXCR2P1 | C-X-C motif chemokine receptor 2 pseudogene | 2 | 2q35 |
| NR_033921 | LOC643542 | uncharacterized LOC643542 | 18 | 18q22.1 |
| XR_109027 | LOC387720 | collagen alpha-1(X) chain | 10 | 10q26.2 |
| NR_028043 | IGF2-AS | IGF2 antisense RNA | 11 | 11p15.5 |
| NM_001164467 | DUX4L3 | double homeobox 4 like 3 | 10,4 | 4q35.2 |
| NR_002835 | HAS2-AS1 | HAS2 antisense RNA 1 | 8 | 8q24.13 |
| NR_038235 | LINC01606 | long intergenic non-protein coding RNA | 8 | 8q12.1 |

**Supplementary Table S2**. Summary of top 10 ranked lncRNAs and their involvement in cancer/disorder.

| **Rank** | **LncRNA-Symbol** | **Cancer/disorder** | **Reference** |
| --- | --- | --- | --- |
| 1 | LOC440896 | Lung carcinoma, breast cancer, pancreatic and glioblastoma | 56-59 |
| 2 | LOC729770 | No literature supported | - |
| 3 | LINC00632 | Breast cancer, prostate cancer, lung carcinoma, medullo blastoma, and glioblastoma | 59-65 |
| 4 | CXCR2P1 | No literature supported | - |
| 5 | LOC643542 | Biopolar disorders, hyperactivity disorder | 66-68 |
| 6 | LOC387720 | No literature supported | - |
| 7 | IGF2-AS | Glioblastoma, ependymoma | 62,71 |
| 8 | DUX4L3 | No literature supported | - |
| 9 | HAS2-AS1 | Breast carcinoma, pancreatic carcinoma, colorectal carcinoma, and non-small cell lung carcinoma | 59, 71 |
| 10 | LINC01606 | Pancreatic adenocarcinoma, oesophageal adenocarcinoma, and hepatobiliary carcinoma | 58, 59, 71, 72 |
